# Systems Pharmacology-Based Method to Assess the Mechanism of Action of Weight-Loss Herbal Intervention Therapy for Obesity

**DOI:** 10.3389/fphar.2019.01165

**Published:** 2019-10-14

**Authors:** Wei Zhou, Ziyi Chen, Yonghua Wang, Xiumin Li, Aiping Lu, Xizhuo Sun, Zhigang Liu

**Affiliations:** ^1 ^Department of Respirology and Allergy, The Third Affiliated Hospital of ShenZhen University, Shenzhen, China; ^2^School of Basic Medical Sciences, Henan University of Traditional Chinese Medicine, Zhengzhou, China; ^3^College of Life Sciences, Northwest University, Xi’an, China; ^4^School of Chinese Medicine, Hong Kong Baptist University, Kowloon, Hong Kong

**Keywords:** obesity, weight-loss herbal intervention therapy (W-LHIT), multi-compounds, systems pharmacology, mechanism

## Abstract

Obesity is a multi-factorial chronic disease that has become a serious, prevalent, and refractory public health challenge globally because of high rates of various complications. Traditional Chinese medicines (TCMs) as a functional food are considered to be a valuable and readily available resource for treating obesity because of their better therapeutic effects and reduced side effects. However, their “multi-compound” and “multi-target” features make it extremely difficult to interpret the potential mechanism underlying the anti-obesity effects of TCMs from a holistic perspective. An innovative systems-pharmacology approach was employed, which combined absorption, distribution, metabolism, and excretion screening and multiple target fishing, gene ontology enrichment analysis, network pharmacology, and pathway analysis to explore the potential therapeutic mechanism of weight-loss herbal intervention therapy in obesity and related diseases. The current study provides a promising approach to facilitate the development and discovery of new botanical drugs.

## Introduction

Obesity is a multi-factor chronic disease involving an abnormal or excessive accumulation of fat in the body. Current trends predict that obesity prevalence rates for the global population will increase to 57.8% by 2030 (Pal et al., 2016). Obesity has become one of the leading health risk factors worldwide because it can induce various complications, particularly cardiovascular diseases, diabetes mellitus, fatty liver, and certain types of cancer (Haslam and James, 2005; Poulain et al., 2006).

The conventional therapeutic approaches for obesity are lifestyle changes, diet restriction, regular physical exercise, bariatric surgery, and pharmacological drugs (Li et al., 2015). However, because of the complex pathological mechanisms underlying obesity and obesity-related diseases, these strategies have proven to be far from satisfactory. Lifestyle changes, diet restriction, and regular physical exercise typically produce modest weight loss. It is a great challenge to sustain long-term behavioral modification, and this may cause unfavorable psychological changes (Li et al., 2015). Surgery is often considered for serious medical conditions, such as those with a high risk of obesity-related diseases and death (Shippey and Macedonia, 2003). In addition, although conventional western anti-obesity drugs are the dominant treatment used by obese patients, they are limited by serious side effects such as negative mood changes, possible liver damage, gastrointestinal or cardiovascular complications, and the potential for drug abuse and dependency for some people. These issues have resulted in a bottleneck in developing a safe and effective weight control strategy.

Given the drawbacks of conventional therapeutic methods, alternative treatments are needed. Traditional Chinese medicines (TCMs) have a long history, and for more than 2000 years, they have been favored by people all over the world for their unique advantages in preventing and treating various diseases and facilitating rehabilitation and health care (Xu and Chen, 2011). Multi-compound and multi-target TCMs have been used by the public at large and provide an alternative treatment for controlling weight and its related symptoms with reputable safety, potential efficacy, low cost, and few adverse effects. It is reported that pioneer investigations concerning clinical studies and animal models have been studied to explore the role of TCM in weight loss (Xiong et al., 2010; Lenon et al., 2012). In a previous study, we developed a TCM formula called weight-loss herbal intervention therapy (W-LHIT) for the prevention of obesity which consists of six herbs: *Ganoderma lucidum* (Ling Zhi), rhizome of *Coptis chinensis* (Huang Lian), *Radix Astragali* (Huang Qi), *Nelumbo nucifera* Gaertn (He Ye), *Chaenomeles speciosa* (Mu Gua), and *Fructus aurantii* (Zhi Qiao). Despite the fact that W-LHIT has been proven to be effective in controlling weight (Yang et al., 2014), it is still difficult to clarify the active compounds, potential targets, the related pathways, and the underlying mechanisms of W-LHIT in the treatment of obesity using traditional experimental methods. Experimental evidence-based studies are not only labor-intensive, costly, and time-consuming but also lack a systematic explanation for the underlying mechanisms of W-LHIT in weight control. Therefore, it is imperative to develop a systematic approach to identify active ingredients and their related targets and clarify the mechanisms of W-LHIT in the treatment of obesity.

Fortunately, systems pharmacology has emerged as a novel strategy to elucidate therapeutic mechanisms and promote drug discovery and development (Zhou et al., 2016). Systems pharmacology involves the dissection of complex interrelationships between compounds, targets, pathways, and diseases at a systems level by combining absorption, distribution, metabolism and excretion (ADME) assessments, multiple drug-target predictions, network pharmacology, and pathway analysis (Zhou et al., 2016). A growing number of evidences suggested that systems pharmacology approaches have been developed to explore the complex mechanism of TCM. For example, a network pharmacology framework was established to translate TCM from an experience-based medicine to an evidence-based medicine system in the past years (Li, 2007; Li and Zhang, 2013). A “network target”–based approach was proposed using network analysis to establish an algorithm termed NIMS (network target–based identification of multicomponent synergy) to screen synergistic drug combinations from TCM herbs or herbal formulae (Li et al., 2011). It is reported that herbal formula Qing-Luo-Yin and Liu-Wei-Di-Huang Pill were used as probes to decipher the combinatorial rule and the pharmacological mechanisms of TCM formulate at the point of network/systemic view (Zhang et al., 2013; Liang et al., 2014). Furthermore, in our previous studies, we have successfully elucidated the underlying mechanisms of TCMs in major types of coronary artery disease and deciphered the mechanisms of botanic drug pairs in treating different diseases based on systems pharmacology methods (Zhou and Wang, 2014; Zhou et al., 2016). The application of systems pharmacology in TCM may permit further understanding of the multiple mechanisms of action of TCMs in treating complex diseases.

**Figure 1:**
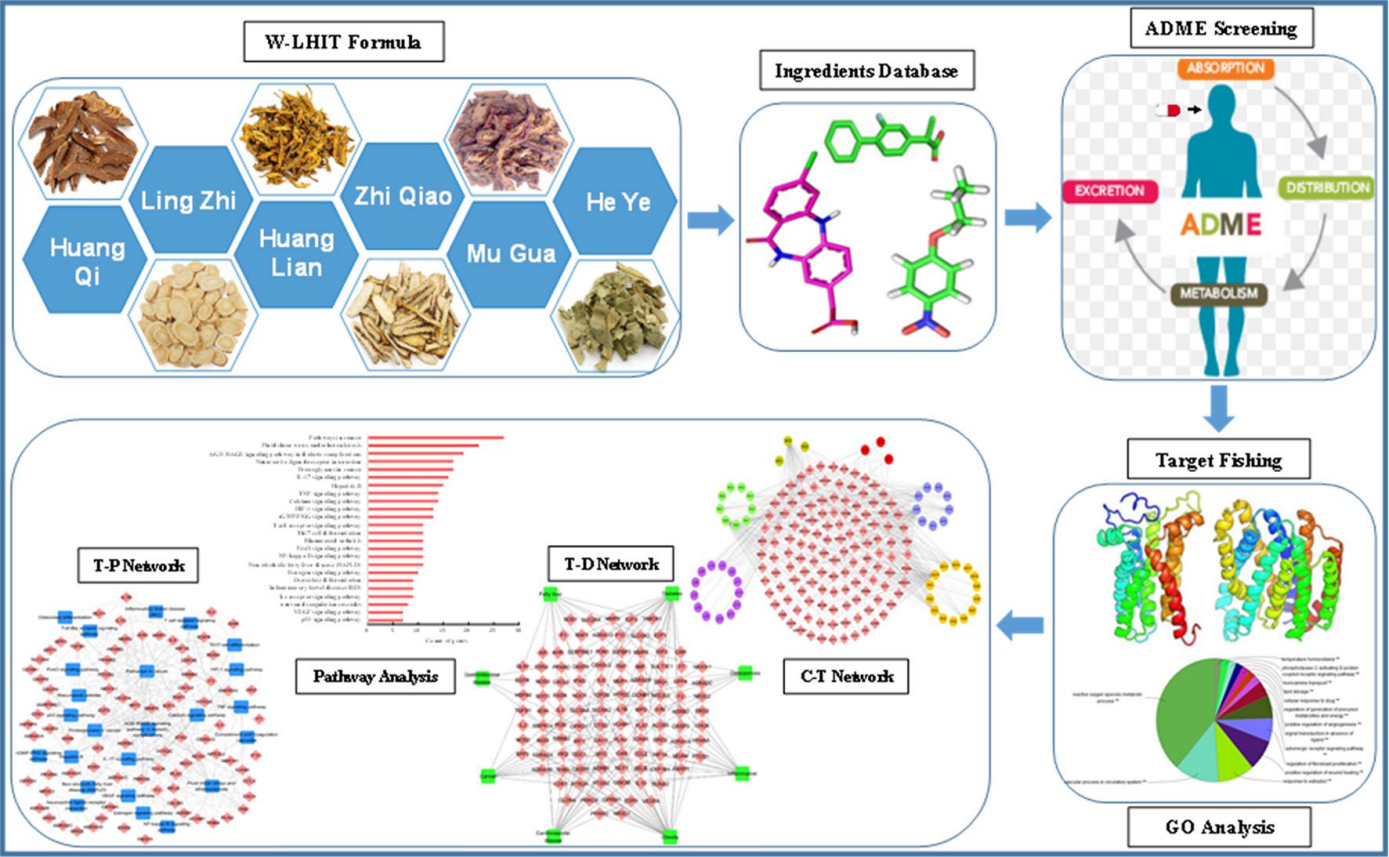
The workflow of the systems pharmacology framework.

Therefore, in the present study, a modified systems-pharmacology framework which integrated an ADME evaluation, herb feature mapping, multiple targeting, gene ontology (GO) enrichment analysis, network pharmacology, and pathway analysis was proposed to clarify the pharmacological mechanism of W-LHIT in the treatment of obesity and related diseases. A comprehensive exploration of W-LHIT based on systems pharmacology not only provides an opportunity to further understand the potential mechanisms of W-LHIT in obesity therapy but also sheds light on a novel method to promote TCM drug discovery for the treatment of complex diseases. A flowchart of the systems pharmacology approach is shown in **Figure 1**.

## Materials and Methods

### Ingredients of Database Construction

The ingredients of the six herbs in the W-LHIT were obtained from the traditional Chinese medicines for systems pharmacology database and analysis platform (Ru et al., 2014), the traditional Chinese medicine integrative database (Xue et al., 2012), the TCM Database @Taiwan (Chen, 2011), and wide-scale literature mining. Finally, 541 chemical compounds from the six herbs in the W-LHIT were listed, including 225 in Ling Zhi, 48 in Huang Lian, 85 in Huang Qi, 87 in He Ye, 80 in Mu Gua, and 16 in Zhi Qiao. These compound structures were saved in mol2 format for further investigation.

### ADME Screening

The major cause of costly late-stage failures during drug development is poor ADME properties (Wang and Urban, 2004). Therefore, ADME evaluation of a given compound in the early stages of drug discovery is extremely important. Multi-component herbal medicine, such as a TCM formula, often contains hundreds or even thousands of ingredients, but only several bioactive compounds produce pharmacological effects in treating disease. The evaluation of pharmacokinetic profiles is a fundamental step for the accurate identification of the active ingredients in TCMs. In recent years, *in silico* methods have become more widely used to find active compounds that possess favorable pharmacokinetic properties, which are useful in assessing the therapeutic mechanism of herbs, since existing biological experimental techniques are generally more labor-intensive, costly, and time-consuming. Therefore, in the current study, two important pharmacokinetic parameters, oral bioavailability (OB) and drug-likeness (DL), were used to screen potential active compounds in W-LHIT.

OB is an important pharmacokinetic indicator in drug screening cascades which represents the fraction of an orally administered dose that enters the systemic circulation to produce a pharmacological effect. In the current study, the OB value was used to identify the active compounds in W-LHIT, and the values were calculated using a robust *in silico* model OBioavail 1.1 (Xu et al., 2012). This model is based on a dataset of 805 structurally diverse drugs and is constructed using the multiple linear regression, partial least squares regression, and support-vector machine (SVR) methods (Hou and Xu, 2002). The SVR as the optimal model exhibits good performance with a determination coefficient (R^2^) of 0.80 and a standard error of estimate of 0.31 for test sets. Finally, the compounds with OB ≥50% were selected as candidate molecules since the values of OB are 30% on average with 10–50% variability based on clinical studies. The threshold determination is mainly based upon two careful considerations: (1) extracting as much as possible from the studied herbs in the W-LHIT with the least number of chemical ingredients; (2) the acquired model can be rationally interpreted using the published pharmacological data.

DL is important in drug design for discriminating “drug-like” molecules from an enormous number of chemical compounds, which helps to optimize pharmacokinetic and pharmaceutical profiles. Therefore, in the current study, a database-dependent model was performed to filter out the drug-like characteristics of the expected molecules from the W-LHIT based on the Tanimoto coefficient (Yamanishi et al., 2010). The formula of the DL index for a new compound is defined as follows:

(1) $$f(A,B)=\frac{A\cdot B}{|A{|}^{2}+|B{|}^{2}-A\cdot B}$$

in which *A* represents the molecular descriptors of the compounds in the herbs, and *B* denotes the average molecular properties of 6,511 structurally diverse drugs and drug-like molecules in the Drugbank database (http://www.drugbank.ca). The compounds meeting the criteria of DL ≥0.18 were selected as potential bioactive compounds because the mean value of the DL index in DrugBank is 0.18 (Liu et al., 2013).

### Multiple Target Fishing

The identification of compound-target interaction profiles has been increasingly necessary to interpret the mechanism of drug action. To predict the targets of active compounds in the herbs, a multiple targeting strategy which effectively integrated a systematic *in silico* prediction model and chemogenomic and data mining was proposed to identify target proteins of the active compounds. Initially, a robust multiple drug-target interaction prediction (DTpre) model that combines chemical, genomic, and pharmacological information based on support vector machine (SVM) and random forest (RF) values was developed to identify the potential drug-target interactions (Yu et al., 2012). The DTpre model performs well in predicting compound-target interactions, with a concordance of 82.83%, sensitivity of 81.33%, and specificity of 93.62%. In the current study, target proteins with SVM values and RF scores larger than 0.8 and 0.7, respectively, were chosen as the final predicted targets. Secondly, the virtual chemical Engerprint Similarity Ensemble Approach (SEA, http://sea.bkslab.org/) was used to identify the targets of active compounds based on the chemogenomic approach (Keiser et al., 2007). Thirdly, text mining was performed using the Therapeutic Target Database (TTD) (http://bidd.nus.edu.sg/group/ttd/) (Chen et al., 2002) and DrugBank (Knox et al., 2010) to extract more accurate interactions between active compounds and targets, and all information was supported by published literature. Finally, to further investigate the mechanisms of W-LHIT in obesity therapy, the final obtained targets were mapped to the Comparative Toxicogenomics Database (CTD, http://ctdbase.org/) (Davis et al., 2012) and TTD to search for related diseases and construct target-disease relationships.

### Gene Ontology Enrichment and Pathway Analysis

To investigate the meaningful biological functional annotation of the potential targets, GO enrichment analysis was introduced to extract the key GO terms (biological process and molecular function) and Kyoto Encyclopedia of Genes and Genomes (KEGG) pathways based on a widely used Cytoscape v2.8.3 plugin ClueGO with a hypergeometric test (Bindea et al., 2009). The target proteins were added to ClueGO in simple text format or interactively derived from gene network graphs visualized in Cytoscape v2.8.3 to symbolize gene function and pathway information. The targets that organized and condensed into several functional groups denoted by their most significant leading term were visualized in the network. The criterion for difference screening included a p-value ≤0.05.

### Network Construction and Analysis

To interpret the pharmacological mechanisms of W-LHIT in obesity at a systems level, a compound-target network (C-T network), target-disease network (T-D network), and target-pathway network (T-P network) were separately established to comprehensively clarify the complicated relationships among the compounds, targets, diseases, and related pathways. The C-T network was constructed by linking the active compounds and their potential targets, and the T-D network was generated by connecting relevant targets with their diseases. The T-P network was constructed by relating the targets to their related biological pathways. In these networks, compounds and targets, diseases, and pathways are marked as nodes, while the interactions between them are represented by edges. All networks were constructed using Cytoscape v2.8.3, which is a powerful bioinformatics package for biological network data visualization, integration, and analysis (Smoot et al., 2010).

## Results and Discussion

### Active Compound Screening for Each Herb in W-LHIT

Generally, many orally administered drugs fail to reach to their target sites because of their poor pharmaceutical properties. Therefore, it is necessary to develop a method to overcome these barriers so as to identify the compounds with satisfactory pharmacokinetic properties. In the current study, two effective *in silico* models (OB and DL) (Yamanishi et al., 2010; Xu et al., 2012) were established to screen the active pharmaceutical compounds in the herbs. As a result, 51 active compounds from the six herbs in the W-LHIT were shown to have satisfactory properties with the filter criterion OB ≥40% and DL ≥0.18 (as shown in **Table S1**). Among them, 13 active compounds with good OB and DL values were from Ling Zhi, such as epoxyganoderiol B (OB = 42.30% and DL = 0.83), ganoderal B (OB = 42.19% and DL = 0.81), and lucialdehyde C (OB = 42.26% and DL = 0.81). For Huang Lian, nine bioactive components met the filter criteria, including corchoroside A (OB = 104.95% and DL = 0.78) and (R)-canadine (OB = 55.37% and DL = 0.77). A total of 14 active molecules were identified in Huang Qi, such as formononetin (OB = 69.67% and DL = 0.21), folic acid (OB = 68.96% and DL = 0.71), and isomucronulatol (OB = 67.67% and DL = 0.26). A total of nine active ingredients were obtained from He Ye, which include machiline (OB = 79.64% and DL = 0.24) and armepavine (OB = 69.31% and DL = 0.29). In addition, only three active compounds were obtained from Mu Gua and Zhi Qiao, respectively. For instance, betulinic acid (OB = 55.38% and DL = 0.78) was identified in Mu Gua, and hesperetin (OB = 70.31% and DL = 0.27) was identified in Zhi Qiao.

Interestingly, of the 51 active ingredients, most have been reported to be associated with various pathological processes including obesity, diabetes, cardiovascular disease, fatty liver, osteoporosis, gastrointestinal disease, inflammation, and cancer. For instance, ergosterol peroxide (OB = 44.39% and DL = 0.82) in Ling Zhi has the potential to suppress lipopolysaccharide (LPS)-induced inflammatory responses by suppressing the transcriptional activity of nuclear factor-κB (NF-κB) and CCAAT-enhancer binding protein β (C/EBPβ) and the phosphorylation of mitogen-activated protein kinases (MAPKs) (Kobori et al., 2007). Obacunone, as one of the oxygenated triterpenoids of Huang Lian, has been confirmed to be beneficial against obesity through the Takeda G-protein receptor 5 (TGR5) and peroxisome proliferator–activated receptor gamma (PPARγ) pathway (Horiba et al., 2015). The protoberberine alkaloid epiberberine (OB = 43.09% and DL = 0.78) from Huang Lian is a potential preventive and therapeutic agent for diabetes (Chen et al., 2018). Calycosin is the major active component in Huang Qi and exhibits beneficial effects against high-fat diet-induced nonalcoholic fatty liver disease (Duan et al., 2018). Betulinic acid (OB = 55.38% and DL = 0.78) may be a promising leading compound for obesity treatment via the regulation of fat and carbohydrate metabolism (de Melo et al., 2009). Moreover, isorhamnetin (OB = 49.60% and DL = 0.31) in He Ye has proved to be a specific antagonistic ligand of PPARγ that may be beneficial in preventing obesity induced by a high-fat diet (Zhang et al., 2016). Kaempferol (OB = 41.88% and DL = 0.24), which is a flavonoid, has been confirmed to be a potential therapy for cardiovascular diseases through inhibiting the migration of vascular smooth muscle cells (Kim et al., 2015). For Mu Gua and Zhi Qiao, the epicatechin (OB = 48.96% and DL = 0.24) in Mu Gua can suppress the expression of adipose tissue CCL19 so as to produce beneficial effects in diet-induced obesity (Sano et al., 2017). Naringenin (OB = 59.29% and DL = 0.21) in Zhi Qiao has shown good pharmacological effects in gastrointestinal disease (Kim and Kim, 2017).

### Target Identification and Analysis

TCM formulas exert their pharmacological activity in various complex diseases through synergistic interactions between multiple compounds and targets. Therefore, the identification of target proteins is necessary in addition to the identification of active compounds. In the current study, several integrated *in silico* approaches, including SysDT, SEA, and TTD were employed to find potential targets of the active compounds in the W-LHIT.

As a result, 111 proteins were identified as the targets of the herbs in the W-LHIT (**Table S2**). The numbers of potential targets affected by active compounds from Ling Zhi, Huang Lian, Huang Qi, He Ye, Mu Gua, and Zhi Qiao are 14, 100, 103, 104, 81, and 15, respectively. Many active compounds exert their pharmacological effects through binding to more than one target simultaneously. For instance, stellasterol from Ling Zhi targets five proteins such as the glucocorticoid receptor (NR3C1). (R)-canadine, as an active molecule in Huang Lian, can interact with 28 targets, including dipeptidyl peptidase IV (DPP4) and peroxisome proliferator–activated receptor gamma (PPARG). Kaempferol (from Huang Qi) was found to be connected with 19 target proteins, such as acetylcholinesterase (ACHE) and prostaglandin G/H synthase 1 (PTGS1), while 27 targets were predicted for armepavine in He Ye, such as the mu-type opioid receptor (OPRM1). For Mua Gua and Zhi Qiao, epicatechin in Mua Gua showed interactions with 10 targets, such as nitric oxide synthase, inducible (NOS2), and naringenin in Zhi Qiao was also linked to 10 targets, such as glycogen synthase kinase-3 beta (GSK3B). The obtained targets may potentially be therapeutic targets for their related diseases. To elucidate the therapeutic mechanism of W-LHIT in various diseases, 111 potential targets were mapped to the PharmGkb, TTD, and CTD database to identify relevant diseases. The interactions between the targets and related diseases are presented in **Table S2**.

### Gene Ontology Enrichment Analysis for Potential Targets

**Figure 2:**
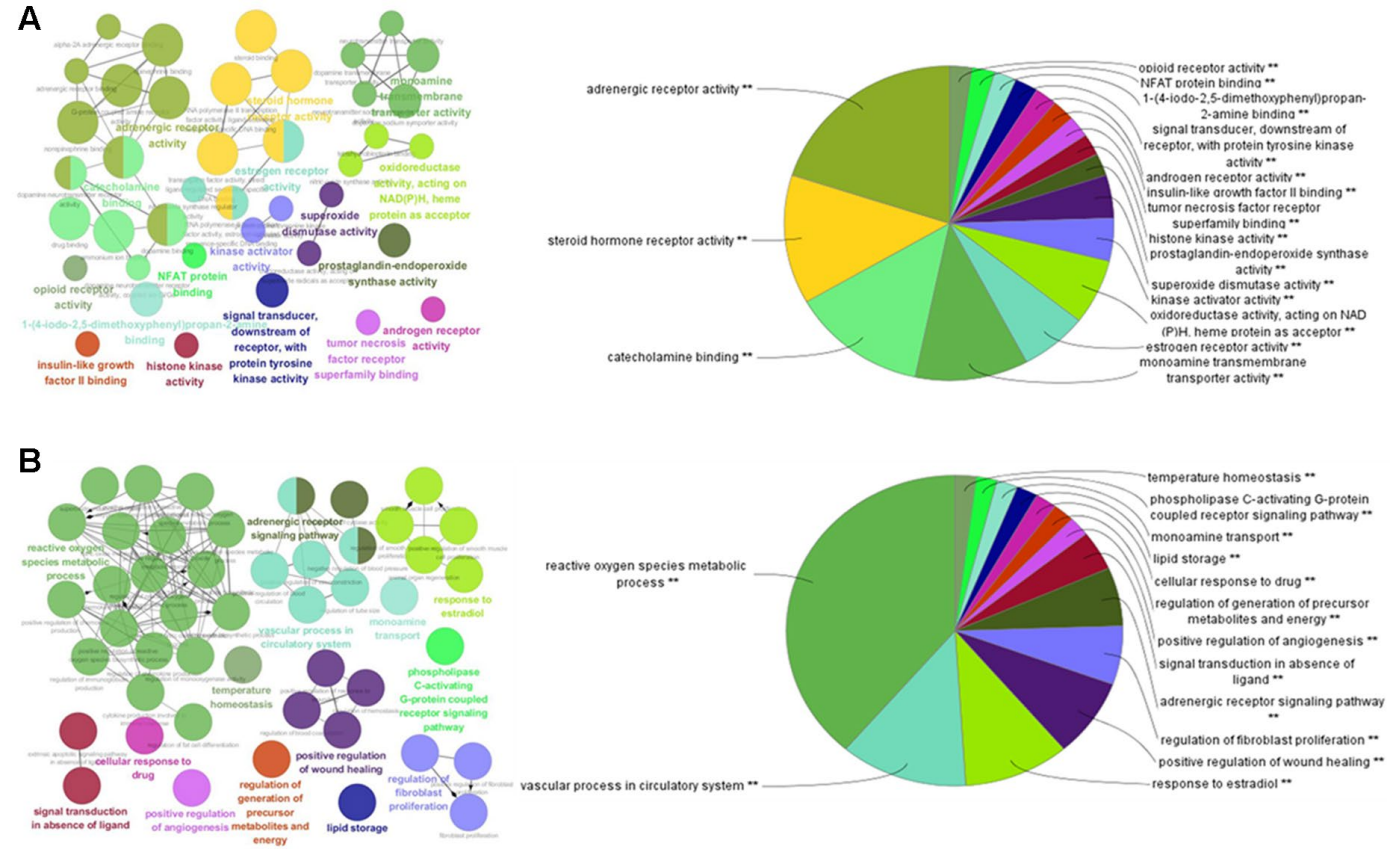
GO enrichment analysis of the predicted protein targets. The nodes represent GO terms with significant enrichment. The node pie charts represent the molecular function and biological process analysis of these targets. **(A)** Representative-enriched molecular function relative to the targets. **(B)** Representative-enriched biological processes relative to the targets.

To further investigate the 111 potential targets in the network, GO term annotations, including molecular function and biological processes, were performed. As shown in **Figure 2A**, the results suggest that the potential targets are involved in various molecular functions which are closely associated with the pathogenesis of obesity and related diseases, such as adrenergic receptor activity, steroid hormone receptor activity, catecholamine binding, monoamine transmembrane transporter activity, estrogen receptor activity, tumor necrosis factor receptor superfamily binding, and insulin-like growth factor II binding. For instance, adrenergic receptor genes play important roles in regulating the lipid mobilization responsible for obesity and diabetes (Takenaka et al., 2012). Steroid hormone receptors, as ligand-dependent intracellular transcription factors, have been reported to be associated with various pathologies such as obesity, diabetes, cardiovascular disease, and inflammation (Kumar, 2016).

For biological process analysis (**Figure 2B**), the top 14 significant GO terms responsible for obesity and related diseases were enriched, including reactive oxygen species metabolic process, the vascular process in circulatory system, the response to estradiol, the regulation of fibroblast proliferation, lipid storage, and the regulation of generation of precursor metabolites and energy. For instance, reactive oxygen species are contributors to oxidative stress that can occur in obesity and related metabolic complications such as diabetes and cardiovascular disease (Le Lay et al., 2014). These results suggest that the targets were enriched and associated with the pathogenesis of obesity and related diseases.

### Network Pharmacology Analysis

Generally, TCM formulas play a potential role in treating various diseases through multiple compounds, targets, and pathways. To elucidate these complex relationships at a systems level, C-T, T-D, and T-P networks were constructed.

#### Compound-Target Network

**Figure 3:**
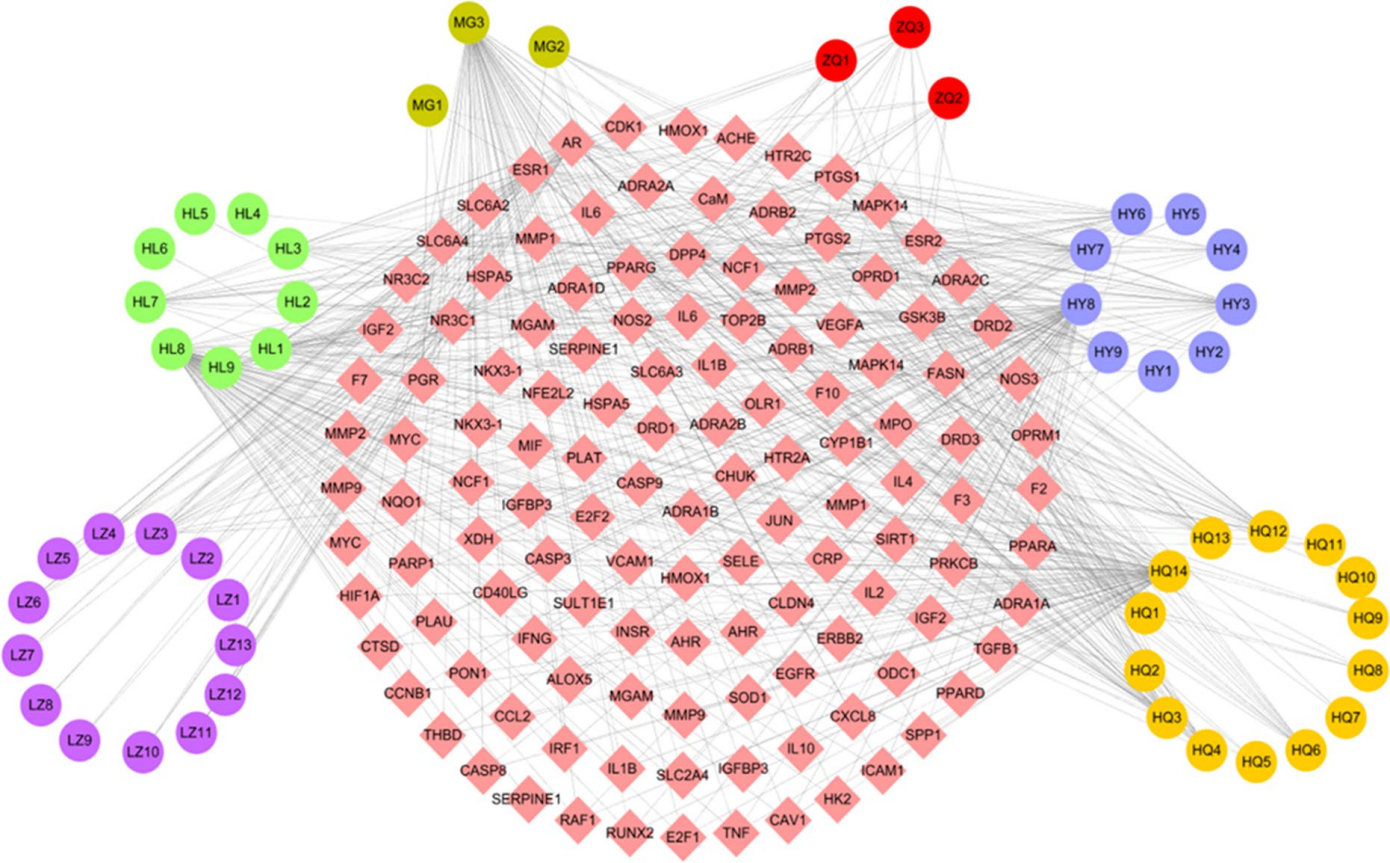
C-T network. The C-T network was constructed by linking active compounds and potential targets. The nodes represent active compounds (circle) and targets (rhombus).

The C-T network consisted of 51 active compounds, 111 targets, and 676 C-T interactions (162 nodes and 676 edges) (**Figure 3**, **Table S2**). This is consistent with the multi-component multi-target characteristics of TCMs. Among the 51 active compounds in the six herbs, 22 demonstrate a high degree and are linked with more than 10 targets. For instance, in Huang Qi, 7 of 14 active compounds exhibit a high degree, including quercetin (degree = 70), 7-O-methylisomucronulatol (degree = 25), 3,9-Di-O-methylnissolin (degree = 20), kaempferol (degree = 19), astrapterocarpan (degree = 16), calycosin (degree = 12), and kumatakenin (degree = 11). Of the nine active molecules, five derived from He Ye: i.e., quercetin (degree = 70), armepavine (degree = 27), machiline (degree = 22), kaempferol (degree = 19), and roemerine (degree = 15). The remaining four compounds derived from Huang Lian, namely, quercetin (degree = 70), (R)-canadine (degree = 28), epiberberine (degree = 12), and palmatine (degree = 16). The two active ingredients in Zhi Qiao showed a high number of interactions with target proteins, including nobiletin (degree = 14) and hesperetin (degree = 11). Quercetin was identified in Mu Gua with a degree of 70, and ergosta-4,6,8 (14),22-tetraene-3-one was identified in Ling Zhi with a degree of 13.

Similarly, the results of a network analysis demonstrated that one target can be targeted by more than one compound from different herbs, which indicates the synergistic effects of TCM formulas. In the C-T network, 64 out of the 111 target proteins exhibited at least 2 interactions with the active compounds of the different herbs. For instance, prostaglandin G/H synthase 2 (PTGS2) is simultaneously targeted by 17 active ingredients from six herbs in the W-LHIT, including five from Huang Qi (3,9-di-O-methylnissolin, 7-O-methylisomucronulatol, astrapterocarpan, calycosin, kaempferol, kumatakenin) and He Ye (armepavine, epicatechin, kaempferol, machiline, roemerine), four from Huang Lian ([R]-canadine, epiberberine, palmatine, worenine), three from Zhi Qiao (hesperetin, naringenin, nobiletin), and one from Mu Gua (epicatechin) and Ling Zhi (ergosta-7,22-dien-3-yl linoleate). These results indicate that the therapeutic effects of W-LHIT in obesity and related diseases likely depend on synergistic interactions between multiple compounds and targets.

#### Target-Disease Network

**Figure 4:**
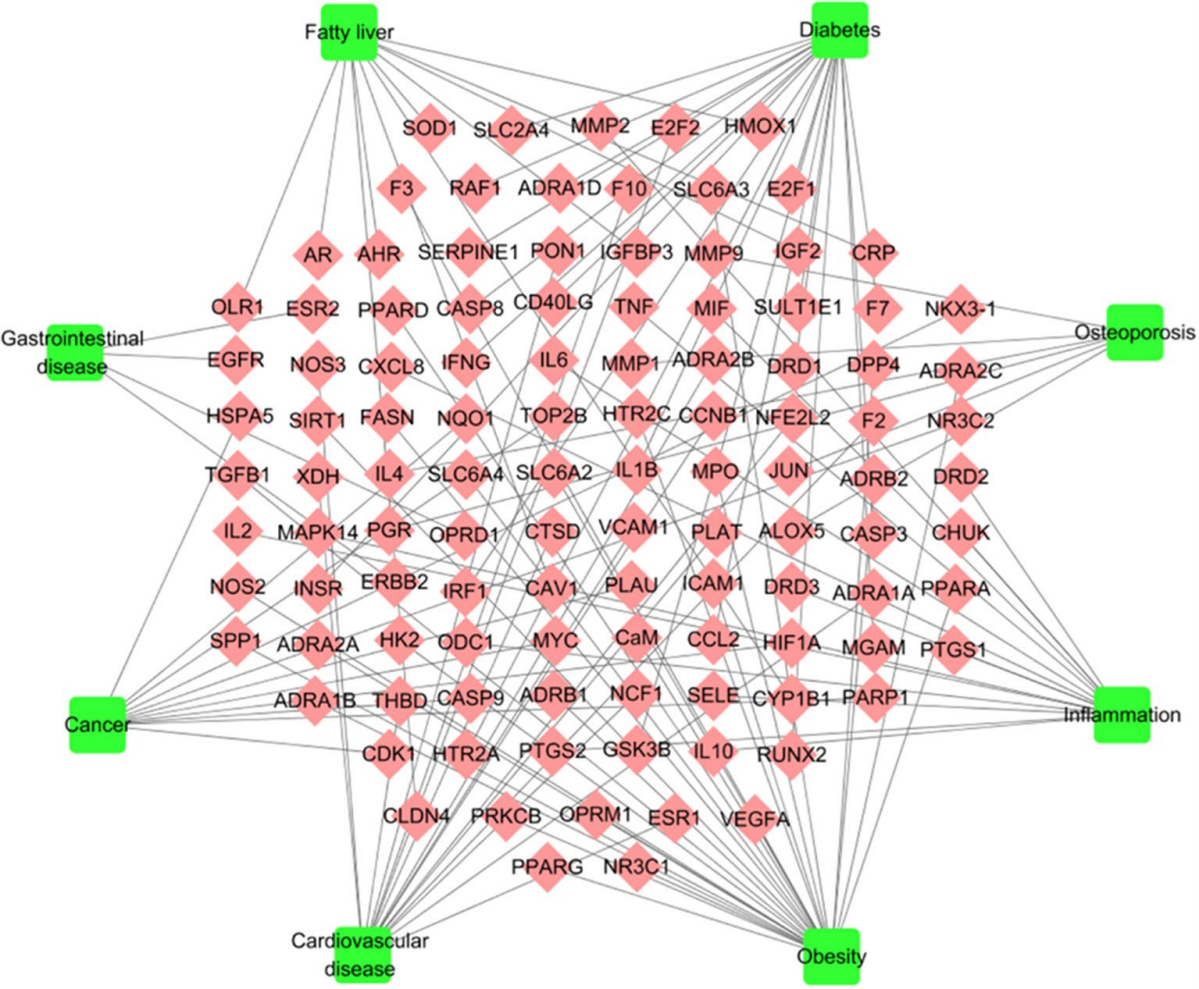
T-D network. The T-D network was constructed by linking potential targets and their related diseases. The nodes represent potential targets (rhombus) and diseases (square).

To explore the pharmacological mechanism of W-LHIT in the treatment of diseases, a T-D network (**Figure 4**, **Table S2**) was constructed by employing 111 target proteins and eight corresponding diseases. As a result, 28 targets (24 in Huang Lian, 21 in Huang Qi, 26 in He Ye, 14 in Mua Gua, 4 in Zhi Qiao, 4 in Ling Zhi) affected by the 6 herbs in the W-LHIT were identified as being closely associated with obesity. For instance, the 5-hydroxytryptamine 2C receptor (HTR2C) targeted by Huang Lian was confirmed as an obesity-related treatment target because of its function in modulating the activity of neuronal pathways regulating energy balance (Burke and Heisler, 2015). PPARG (targeted by Huang Qi) is involved as a nuclear receptor in the pathological process of obesity (Motawi et al., 2017). It has been suggested that fatty acid synthase (FAS) is a potential therapeutic target for anti-obesity drugs with the highest level of enriched expression in human adipocytes (Liang et al., 2018). NR3C1, which is targeted by He Ye, is related to the etiology of obesity, the control of which will lead to an improvement in obesity-related disorders (Majer-Łobodzińska and Adamiec-Mroczek, 2017). Moreover, DDP4 (targeted by Zhi Qiao) and ACHE (targeted by Ling Zhi) play a significant role in obesity, and modulating their activity may be of great utility in obesity treatment (Valerio et al., 2017; Shenhar-Tsarfaty et al., 2019).

Obesity is a complex disease that often leads to other diseases. In the current study, 17, 20, 14, 4, 6, 15, and 11 target proteins are considered to have significant relationships with the pathological processes of cardiovascular diseases (15 in Huang Lian, 14 in Huang Qi, 16 in He Ye, 13 in Mua Gua, 3 in Zhi Qiao, 3 in Ling Zhi), diabetes (20 in Huang Lian, 20 in Huang Qi, 19 in He Ye, 16 in Mua Gua, 2 in Zhi Qiao, 1 in Ling Zhi), fatty liver (10 in Huang Lian, 11 in Huang Qi, 11 in He Ye, 10 in Mua Gua, 1 in Zhi Qiao, 1 in Ling Zhi), gastrointestinal disease (4 in Huang Lian, 4 in Huang Qi, 4 in He Ye, 3 in Mua Gua, 1 in Zhi Qiao), osteoarthritis (4 in Huang Lian, 6 in Huang Qi, 5 in He Ye, 5 in Mua Gua, 1 in Ling Zhi), inflammation (13 in Huang Lian, 12 in Huang Qi, 13 in He Ye, 11 in Mua Gua, 3 in Zhi Qiao, 2 in Ling Zhi), and cancer (10 in Huang Lian, 10 in Huang Qi, 10 in He Ye, 9 in Mua Gua, 1 in Zhi Qiao, 1 in Ling Zhi), respectively. This implies that W-LHIT may exert pharmacological effects not only in obesity but also in these related diseases.

For instance, nitric oxide synthase, endothelial (NOS3), targeted by Huang Lian has been reported to be a mediator of angiogenesis that is responsible for pathological processes in cardiovascular disease (Wang et al., 2015). It has been demonstrated that GSK3B (targeted by Huang Qi) is a novel target for the treatment of diabetes mellitus (Gao et al., 2012). He Ye was found to act on insulin-like growth factor II (IGF2), transient overexpression of which can cause fatty liver disease with the accumulation of free cholesterol, phospholipids, and lipid droplets (Kessler et al., 2016). Estrogen receptor beta (ESR2), which is targeted by Zhi Qiao, is expressed in the gastrointestinal tract and provides a therapeutic strategy to treat patients suffering from gastrointestinal diseases induced by excessive neuronal/glial cell damage (D’Errico et al., 2018). Compounds in Mu Gua target tumor necrosis factor (TNF), which is a well-known cytokine involved in inflammatory processes (López-Urrutia et al., 2017). The progesterone receptor (PGR) is targeted by Ling Zhi and plays a role in reducing the risk of osteoporosis (Zhong et al., 2017). Overall, the complicated interactions between multiple targets and diverse diseases achieved with TCM formulations imply that the synergistic and therapeutic effects of such an approach are better than highly targeted drugs in isolation.

#### Target-Pathway Network

**Figure 5:**
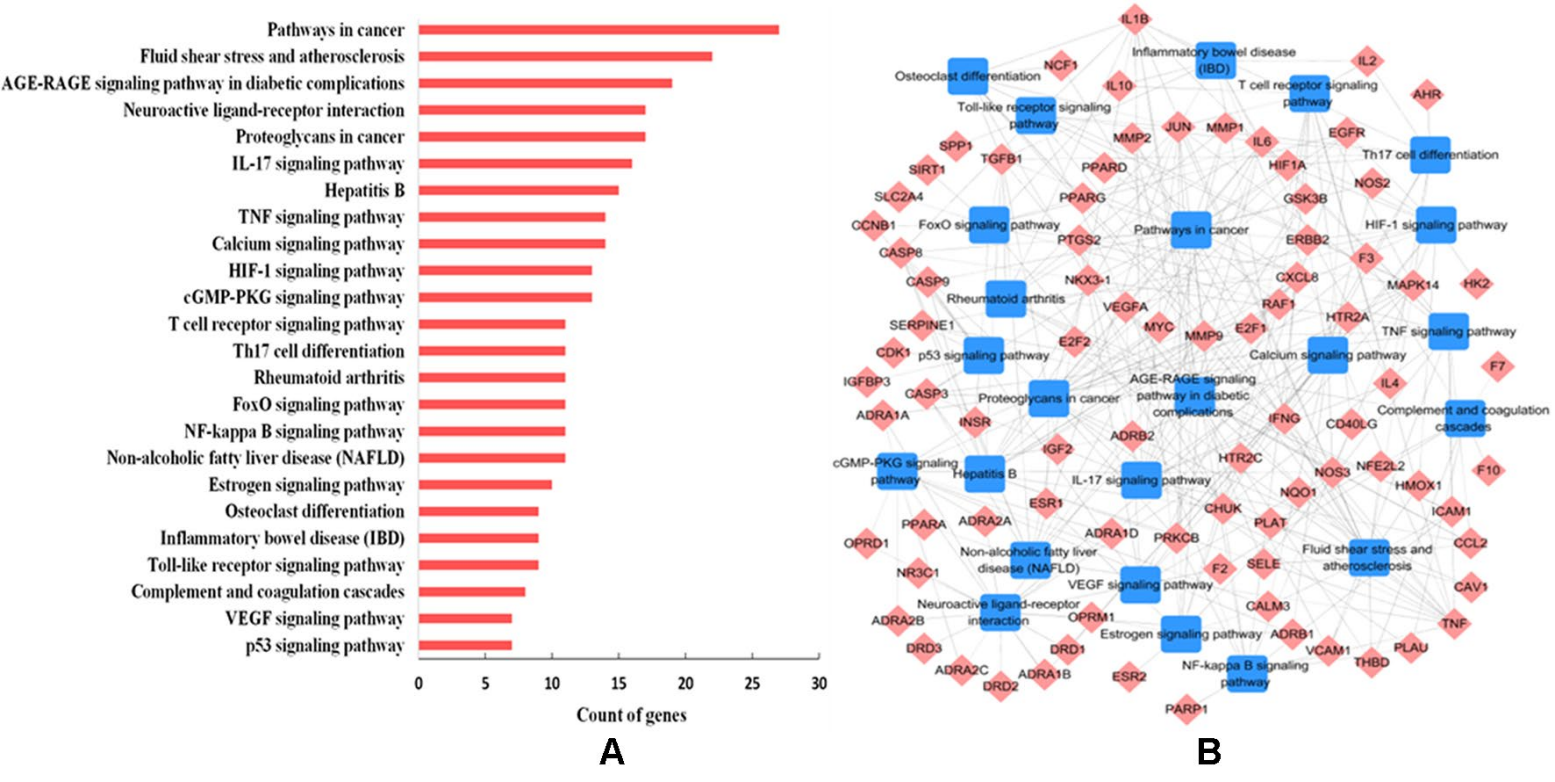
**(A)** Pathway analysis of the targets. The *y*-axis represents the name of significantly enriched pathways related to the target genes, and the *x*-axis represents the target counts. **(B)** T-P network. The T-P network was constructed by linking active compounds and their related pathways. The nodes represent active compounds (rhombus) and pathways (square).

To further decipher the underlying therapeutic mechanisms of W-LHIT for the treatment of obesity and related diseases, all predicted target proteins were mapped onto ClueGO to enrich their relevant pathways. As a result, 24 KEGG pathways were obtained including fluid shear stress and atherosclerosis, the AGE-RAGE signaling pathway in diabetic complications, neuroactive ligand-receptor interaction, the IL-17 signaling pathway, the TNF signaling pathway, and the NF-kappa B signaling pathway (**Figure 5A**). The T-P network containing 111 targets and 24 corresponding pathways is shown in **Figure 5B**.

The results suggest that the enriched pathways are closely related to various pathological processes such as obesity, cardiovascular disease, diabetes mellitus, fatty liver, osteoporosis, inflammation, and certain types of cancer. For example, 27 targets are involved in mediating the pathways in cancer, the dysregulation of which is recognized as a diagnostic marker for various types of cancer (Koury et al., 2017). The 22 targets involved in fluid shear stress and atherosclerosis take part in the regulation of atherosclerosis, which is a major pathogenic factor in cardiovascular diseases (Baeyens et al., 2016). The AGE-RAGE signaling pathway (involving 19 targets) is a well-studied cascade and has been shown to play a role in the maintenance and regulation of the extracellular matrix in diabetes (Kay et al., 2016). A total of 16 targets participate in the IL-17 signaling pathway, which is a novel therapeutic target for the treatment of nonalcoholic fatty liver disease *via* the modulation of hepatocellular damage (Harley et al., 2014). The TNF signaling pathway (involving 14 targets) is one of the most studied pathways involved in the regulation of the inflammatory response (López-Urrutia et al., 2017).

Taken together, these results suggest that multiple targets could affect various pathways responsible for regulating the pathologic processes underlying obesity and related diseases, modulation of which may be a potent therapeutic approach for these diseases.

## Conclusion

Obesity is considered a metabolic disease characterized by an excess storage of body fat and has become a serious growing public health problem globally because of high rates of various complications including cardiovascular disease, diabetes, fatty liver, osteoarthritis, gastrointestinal disease, inflammation, and cancer. TCM has always been regarded as an alternative therapy that has been used to prevent and treat various diseases for thousands of years. However, the potential mechanisms of TCM in obesity have not been fully elucidated. To clarify the pharmacological mechanisms of W-LHIT in obesity and related diseases, an integrated systems pharmacology model that incorporates ADME screening, target prediction, GO enrichment analysis, network technology, and pathway analysis was employed.

Through ADME screening, 51 active compounds with satisfactory pharmacokinetic properties were identified from the six herbs in the W-LHIT. These active compounds could bind to 111 target proteins involved in pathologic processes underlying obesity, cardiovascular disease, diabetes, fatty liver, osteoarthritis, gastrointestinal disease, inflammation, and cancer, suggesting that the herbs may exert pharmacological effects by regulating these targets. After target identification, GO enrichment analysis was performed for molecular function and biological processes to uncover a significant biological functional annotation for the obtained targets. Moreover, the constructed C-T, T-D, and T-P networks suggest that the herbs in W-LHIT play a significant role not only in obesity but also in treating related complications, which reveals that multiple diseases may be treated using a common herbal medicine. Furthermore, the pathway analysis suggests that the six herbs may simultaneously target several related signaling pathways, demonstrating the synergistic mechanism of W-LHIT in the treatment of obesity and related diseases at the pathway level.

In conclusion, the current study has provided a systems pharmacology framework to identify active compounds and potential target proteins and elucidate the pharmacological mechanism of W-LHIT for the treatment of obesity and related diseases. The present work offers a novel and reliable strategy to investigate the complex therapeutic mechanism of W-LHIT in obesity and related diseases at a systems level, which has the potential to facilitate drug discovery using TCMs and the identification of treatments for other complex diseases.

## Data Availability Statement

The raw data supporting the conclusions of this manuscript will be made available by the authors, without undue reservation, to any qualified researcher.

## Author Contributions

ZL and WZ conceived the research; WZ and ZC designed the research, analyzed the data and wrote the paper; WZ and YW developed the theoretical models and performed the computation; XL, AL and XS critically discussed the manuscript. All authors have read and approved the final manuscript.

## Conflict of Interest

The authors declare that the research was conducted in the absence of any commercial or financial relationships that could be construed as a potential conflict of interest.

## Supplementary Material

Table S1 The information of 51 active compounds in W-LHIT.

Table S2 The interactions between the targets and related diseases.
